# ICU admission delays: Impact on length of stay and long-term outcomes

**DOI:** 10.17305/bb.2025.12888

**Published:** 2025-09-26

**Authors:** Ferhan Demirer Aydemir, Ozge Kurtkulagi, Bisar Ergun, Vecihe Bayrak, Ozlem Oner, Bilgin Comert, Ali Necati Gokmen, Volkan Hanci

**Affiliations:** 1 Department of Internal Medicine, Division of Intensive Care Medicine, Çanakkale Onsekiz Mart University Hospital, Çanakkale, Türkiye; 2 Department of Internal Medicine, Faculty of Medicine, Çanakkale Onsekiz Mart University, Çanakkale, Türkiye; 3 Department of Internal Medicine, Division of Intensive Care Medicine, Dokuz Eylul University Faculty of Medicine, Izmir, Türkiye; 4 Department of Anesthesiology and Reanimation, Division of Intensive Care Medicine, Dokuz Eylul University Faculty of Medicine, Izmir, Türkiye

**Keywords:** ICU admission delay, length of stay, Charlson Comorbidity Index, APACHE-II, triage, long-term outcomes

## Abstract

Delays in intensive care unit (ICU) admissions are prevalent in overcrowded hospitals and can adversely affect patient outcomes. However, the extent of this impact, particularly beyond short-term mortality, remains unclear. We hypothesized that ICU admission delays exceeding 6 h after consultation would independently increase 90-day mortality and prolong ICU length of stay. We conducted a retrospective analysis of data from 273 adult patients admitted to the ICU of a tertiary university hospital between January and December 2019. Patients were stratified into two groups: early admission (≤6 h) and delayed admission (>6 h). Multivariate Cox regression was employed to identify independent predictors of mortality. Delayed ICU admission was observed in 72.8% of patients. Although delayed admission was not independently associated with increased mortality in the multivariate analysis (HR: 0.88; 95% CI: 0.61–1.27), it was significantly correlated with prolonged ICU length of stay and higher 90-day mortality in the univariate analysis (*P* ═ 0.039), with no significant difference in vasopressor-free days (*P* ═ 0.809). In our assessment of independent mortality predictors, we found that patients with higher APACHE-II and Charlson scores experienced longer delays in ICU transfer. Additionally, respiratory and circulatory failure at admission were independently associated with increased mortality (HR: 2.17; 95% CI: 1.51–3.12). While early ICU admission did not independently predict mortality, it was linked to extended ICU stays, an increased treatment burden, and adverse long-term outcomes. These findings underscore the necessity of refining triage processes and evaluating baseline patient severity when interpreting the impact of ICU admission timing on outcomes.

## Introduction

The global shortage of intensive care unit (ICU) beds poses a significant challenge, especially amidst increasing demand for critical care and a growing geriatric population. Although limited bed availability is commonly cited as the primary reason for non-admission to ICUs, other factors—such as misconceptions about ICU benefits, inadequate triage practices, and physician decision-making—further complicate access to timely intensive care [1]. The uneven distribution of ICU resources exacerbates delays in admissions, which can adversely affect patient outcomes.

To inform equitable health policy and resource allocation, evidence-based benchmarks for ICU capacity have been proposed. High-income countries are advised to maintain 5–7 ICU beds per 100,000 population to adequately meet demand and ensure surge capacity, while low- and middle-income countries often fall below 2 beds per 100,000. The COVID-19 pandemic highlighted these vulnerabilities; a population-based cohort study in Japan revealed that lower regional ICU capacity was significantly associated with higher rates of mechanical ventilation and mortality during SARS-CoV-2 outbreaks [2]. These findings underscore the critical importance of strategic ICU planning in enhancing preparedness for future infectious disease crises.

The COVID-19 pandemic brought this issue into sharp focus worldwide. Even in high-income countries such as Italy, shortages of ICU beds were linked to increased morbidity and overwhelmed healthcare systems [3]. In many hospitals, critically ill patients experience delays in ICU transfer, which can postpone life-sustaining therapies and increase reliance on invasive interventions [4]. While timely ICU admission has been correlated with improved patient outcomes, delays are often viewed as unavoidable due to logistical constraints and systemic inefficiencies [5].

For patients requiring ventilatory support, ICU admission is ideally recommended within one hour [6]. However, actual waiting times can range from 2 h to 3.5 days, depending on institutional capacity [5, 7–10]. Although prior studies have primarily examined the relationship between delayed admission and mortality, fewer investigations have focused on its impact on ICU resource utilization, including length of stay and duration of organ support. In this study, we define delay as the time from the ICU consultation request to the actual ICU transfer, marking the clinical decision point for ICU need, independent of prior delays in diagnosis or ward-level management.

We hypothesize that delays in ICU admission exceeding 6 h after consultation will independently increase 90-day mortality and prolong ICU length of stay.

## Materials and methods

### ICU setting

This study analyzes a multidisciplinary mixed medical-surgical ICU situated within a tertiary university hospital. The unit has a total capacity of 22 beds and admits approximately 2000 critically ill patients annually, with occupancy rates frequently surpassing 90%. The ICU accommodates both emergency and ward-based admissions and is collaboratively managed by the Departments of Internal Medicine and Anesthesiology.

### Population

This retrospective cohort study comprised patients evaluated for hospitalization in the multidisciplinary ICU of the Departments of Anesthesiology and Internal Medicine at a tertiary university hospital from January to December 2019, all of whom met the criteria for ICU admission. Patients under 18 years of age were excluded from the analysis. The medical records of all ICU admissions were systematically reviewed using a standardized case report form.

### Population characteristics, disease severity assessment, and clinical outcomes

Age, gender, comorbidities (including hypertension, diabetes mellitus, respiratory failure, chronic obstructive pulmonary disease, coronary artery disease, heart failure, renal failure, liver disease, solid organ malignancies, and hematologic malignancies), illness severity scores (APACHE-II, SOFA), clinical characteristics (such as respiratory and other organ failures, non-invasive and invasive ventilation support, duration of mechanical ventilation, vasopressor use, Charlson Comorbidity Index, and the need for hemodialysis), laboratory findings, consultation date and time, response date and time, ICU admission date and time, admission waiting time, admission unit, length of hospital stay, ICU length of stay, and 28-day and 90-day mortality were extracted from medical records.

“Ventilator-free days” and “vasopressor-free days” were defined as the number of days during the ICU stay when the patient was alive and not receiving the respective support. This ICU-specific definition was consistently applied across all analyses and tables to clearly differentiate the defined follow-up for survival analysis from the total duration of hospitalization and treatment.

Patients admitted to the ICU were categorized into two groups: those with a waiting time of 6 h or more (delayed admission) and those admitted within 6 h (early admission). The choice of 6 h as the cutoff for early versus late ICU admissions is supported by previous studies indicating that patients in the U.S. typically spend an average of at least 6 h in non-ICU areas before receiving ICU-level care. Additionally, this threshold aligns with the average wait time of 5.8 h in the emergency department for an acute or critical care bed in overcrowded hospitals, as reported by the American Hospital Association [8]. This 6-h period is also associated with poorer outcomes for patients requiring transfer from the ward to the ICU [11].

### Primary and secondary endpoints

The primary objective of this study was to evaluate the relationship between delayed ICU admission—defined as admission occurring more than six hours after consultation—and both the length of ICU stay and long-term outcomes, specifically 90-day mortality.

The secondary objectives included:

● Examining the association between delayed ICU admission and the duration of mechanical ventilation and vasopressor support;

● Assessing the effect of admission timing on 28-day mortality;

● Analyzing the distribution of delayed versus early admissions by time of day (on-hours versus off-hours);

● Investigating the correlation between delays in ICU admission and illness severity scores (APACHE-II and Charlson Comorbidity Index);

● Evaluating the impact of admission delays on ICU resource utilization and patient throughput.

These objectives were chosen to enhance our understanding of how delayed ICU transfer influences not only survival rates but also the intensity and duration of care required, which directly impacts ICU burden and hospital efficiency.

### Ethical statement

Ethical approval for this retrospective cohort study was granted by the Ethics Committee of Dokuz Eylul University Faculty of Medicine, Türkiye, on January 20, 2020 (Approval No: 2020/01-14). The research was conducted in the ICU of this tertiary university hospital and adhered to the principles of the Helsinki Declaration, as revised in 2013.

### Statistical analysis

Statistical analysis was performed using SPSS (Statistical Package for the Social Sciences Version 24; IBM Corporation, Armonk, NY, USA). Categorical variables were reported as frequencies and percentages, while continuous variables were expressed as medians and interquartile ranges. Comparisons of categorical variables between groups were conducted using the chi-square test or Fisher’s exact test, and continuous variables were analyzed using the Mann–Whitney *U* test. The independent effect of ICU admission timing on mortality was evaluated through multivariate Cox regression analysis. A purposive selection method, adjusted for confounders and statistical significance, was employed to identify a subset of clinically relevant covariates for model development.

Two distinct multivariate Cox regression models assessed the impact of ICU admission delay on 28-day and 90-day mortality. In addition to the primary binary classification of ICU admission delay (≤6 h vs >6 h), an exploratory subgroup analysis was conducted, stratifying ICU admission waiting time into three intervals: ≤2 h, 2–4 h, and >4 h. This post hoc categorization aimed to visually explore potential non-linear associations between waiting time and survival outcomes, although it was not included in the original analysis plan and should be interpreted as exploratory.

Model 1 incorporated age, gender, Charlson Comorbidity Index, comorbidities, admission diagnosis, and ICU admission timing. To assess the robustness of the effect estimate, Model 2 excluded age and comorbidities. In Model 1, independent variables included gender (female/male), presence of comorbidities (<2 vs ≥3), hospitalization diagnoses (respiratory and circulatory failure vs other diagnoses), and hospitalization duration (<6 h vs ≥6 h). Age and the Charlson Comorbidity Index were treated as continuous variables. Due to the established relationship between the Charlson Comorbidity Index and diagnoses, age was removed in Model 2. Adjusted hazard ratios (HR) with 95% confidence intervals (CI) were reported for each independent factor.

Prior to multivariable modeling, multicollinearity among covariates was assessed using Variance Inflation Factor (VIF) diagnostics. All included variables had VIF values below 2.5, indicating no significant multicollinearity. The Charlson Comorbidity Index (CCI) was retained as a composite measure of comorbidity burden due to its established clinical utility. Sensitivity analyses modeled the CCI categorically to evaluate potential non-linear effects. The proportional hazards assumption for Cox regression analyses was tested using Schoenfeld residuals and log–log survival plots, revealing no significant violations (Table S1). Influence diagnostics did not identify any outliers that materially affected model estimates. VIF calculations for covariates ranged from 1.0 to 1.8 in Model 1 and from 1.0 to 1.02 in Model 2, all well below the conventional threshold of 2.5, confirming the absence of significant multicollinearity. A *P* value of <0.05 was considered statistically significant.

### Handling of missing data

Missingness was minimal (<5%) and primarily pertained to laboratory variables. Consequently, we employed a complete-case analysis without imputation for the primary models. As a sensitivity analysis, we repeated the multivariable analyses by incorporating indicator terms for variables with missing data; the results remained largely unchanged (data available upon request).

### Sample size and power analysis

We conducted a post hoc power analysis using G*Power 3.1, employing a Cox proportional hazards model with a two-tailed significance level of α ═ 0.05. The total sample size was *N* ═ 273, with an allocation ratio of delayed to early ICU admission of 199 to 74 (approximately 2.69), and an overall 90-day event proportion of approximately 0.62. For the reference effect size, we utilized the findings from Sabaz et al., which reported increased mortality associated with delayed ICU admission, with hazard ratios ranging from 1.55 to 2.29 across various delay intervals.

Based on these parameters, the achieved power (1–β) to detect a hazard ratio (HR) of 1.50 was approximately 0.65, for HR = 1.60 it was approximately 0.78, for HR = 1.70 it was approximately 0.87, and for HR = 2.00 it was approximately 0.98. The minimum detectable HR at 80% power was approximately 1.62. These calculations suggest that our study is adequately powered to identify moderate to large effects on 90-day mortality, consistent with existing literature, while smaller effects (HR≈1.5) may be underpowered.

## Results

### Patients

A total of 273 patients (54.9% male) were included in the analysis, all of whom were evaluated for ICU admission and had an indication for hospitalization. The median age of the cohort was 72 years (range: 64–81). Of these patients, 51.3% were admitted from general wards, 46.5% from the emergency department, and 2.2% from other intensive care units. The primary diagnoses at the time of hospitalization included respiratory failure in 250 patients (91.6%) and circulatory failure in 133 patients (48.7%). Among female patients (*n* ═ 123, 45.1%), the median age was also 72 years (IQR: 63–84), with the majority being admitted from general wards (56.1%) or the emergency department (42.3%) (Table 1).

**Table 1:** Baseline demographic and clinical characteristics of the study population (total *n* ═ 273)

**Variable** **continuous variables (median, IQR)**	**Overall (*n* ═ 273)**	**Female (*n* ═ 123, 45.1%)**
Age (years)	72 (64–81)	72 (63–84)
Glasgow coma score	4 (3–15)	13 (7–15)
APACHE-II score	24 (17–30)	19 (12–27)
Charlson comorbidity index	5.9 (4–8)	5 (3–7)
Ventilator-free days	1.3 (0–1)	1 (0–3)
Vasopressor-free days	10.8 (0–10)	4 (0–9)
Male gender, *n* (%)	150 (54.9)	
*Comorbidities*		
Hypertension, *n* (%)	140 (51.3)	67 (54.5)
Diabetes mellitus, *n* (%)	92 (33.7)	44 (35.8)
Coronary artery disease, *n* (%)	77 (28.2)	23 (18.7)
Congestive heart failure, *n* (%)	60 (22.0)	28 (22.8)
Peripheral artery disease, *n* (%)	13 (4.8)	5 (4.1)
COPD, *n* (%)	55 (20.1)	24 (19.5)
Dementia, *n* (%)	34 (12.5)	12 (9.8)
Acute kidney failure, *n* (%)	20 (7.3)	11 (8.9)
Chronic kidney failure, *n* (%)	50 (18.3)	15 (12.2)
Liver disease, *n* (%)	16 (5.9)	7 (5.7)
Solid organ malignancy, *n* (%)	58 (21.2)	23 (18.7)
Hematologic malignancy, *n* (%)	34 (12.5)	22 (17.9)
Cerebrovascular disease, *n* (%)	32 (11.7)	16 (13.0)
Connective tissue disease, *n* (%)	19 (7.0)	10 (8.1)
Neuromuscular disease, *n* (%)	3 (1.1)	3 (2.4)
Epilepsy, *n* (%)	0 (0)	0 (0)
Other comorbidities, *n* (%)	110 (40.3)	48 (39.0)
*Diagnosis on admission*		
Respiratory failure, *n* (%)	250 (91.6)	108 (87.8)
Circulatory failure, *n* (%)	133 (48.7)	58 (47.2)
Sepsis, *n* (%)	50 (18.3)	23 (18.7)
Cerebrovascular event, *n* (%)	11 (4.0)	6 (4.9)
Trauma, *n* (%)	6 (2.2)	2 (1.6)
Postoperative, *n* (%)	29 (10.6)	14 (11.4)
CPR, *n* (%)	25 (9.2)	9 (7.3)
Need for hemodialysis, *n* (%)	80 (29.3)	35 (28.5)
Mortality, *n* (%)	174 (63.7)	70 (56.9)
28-day mortality, *n* (%)	124 (45.4)	74 (60.2)
90-day mortality, *n* (%)	169 (61.9)	57 (46.3)
Admission site – ED, *n* (%)	127 (46.5)	52 (42.3)
Admission site – Ward, *n* (%)	140 (51.3)	69 (56.1)
Admission site – Other ICU, *n* (%)	6 (2.2)	2 (1.6)

Note: This table includes overall values and an additional female-only column. All categorical variables were expressed as numbers and percentages; continuous variables were expressed as median IQR. *Other comorbidities: hematologic malignancies, connective tissue diseases, benign prostatic hyperplasia, heart valve disorders, heart rhythm disorders, osteoporosis. Abbreviations: GCS: Glasgow coma scale; CPR: Cardiopulmonary resuscitation; HD: Hemodialysis; ICU: Intensive care unit.

### Comorbidities

The most prevalent comorbidities among patients were hypertension (51.3%), diabetes mellitus (33.7%), coronary artery disease (28.2%), congestive heart failure (22%), and solid organ malignancy (21.2%). The median Charlson Comorbidity Index score at admission was 5.9 (range: 4–8). In the female subgroup, the most common comorbidities included hypertension (54.5%), diabetes mellitus (35.8%), and congestive heart failure (22.8%), with a median Charlson Index of 5 (IQR: 3–7) (Table 1).

### Illness severity status

At admission, the median Glasgow Coma Scale (GCS) score was 4 (range: 3–15), and the median APACHE-II score was 24 (range: 17–30). Hemodialysis was required for 80 patients (29.3%) during their stay in the ICU. The mean duration without mechanical ventilation was 1.3 days (range: 0–1), while the mean duration without vasopressor support was 10.8 days (range: 0–10). In female patients, the median GCS was 13 (IQR: 7–15), the median APACHE-II score was 19 (IQR: 12–27), and 28.5% required hemodialysis during their ICU stay. The median number of mechanical ventilation-free days was 1 (IQR: 0–3), and the median number of vasopressor-free days was 4 (IQR: 0–9) (Table 1).

### Primary results

A statistically significant difference in the length of ICU stay was observed between the early (≤6 h) and delayed (>6 h) ICU admission groups, with median stays of 13.08 days and 16.91 days, respectively (*P* ═ 0.035) (Table 2). This finding supports the primary hypothesis that delayed ICU admission is associated with prolonged ICU stays.

**Table 2:** Comparison of demographic and clinical characteristics between early (≤6 h) and delayed (>6 h) ICU admission groups

**Variables**	**Early admission (≤6 h)** **(*n* ═ 74)**	**Late admission (>6 h)** **(*n* ═ 199)**	***P* value**
Age	68 (57–84)	70 (65–80)	0.821
Glasgow coma scale	10 (3–15)	7 (3–14)	**<0.001**
Charlson comorbidity index	5.78 (3–8)	6.01 (4–8)	0.556
Gender (male)	36 (48.6%)	113 (56.8%)	0.383
*Admission diagnosis*			
Respiratory failure	61 (82.4%)	189(94.9%)	0.002
Circulatory failure	42 (56.7%)	91 (45.7%)	0.134
Sepsis	15 (20.2%)	35 (17.5%)	0.602
Cerebrovascular event	7 (9.4%)	25 (12.5%)	0.534
Trauma	2 (2.7%)	4 (2.0%)	0.664
Postoperative	7 (9.4%)	22 (11.0%)	0.827
CPR	6 (8.1%)	19 (9.5%)	0.817
Ventilator-free days(median, IQR)	1.96 (0–2.3)	1.06 (0–1)	0.003
Vasopressor-free days (median, IQR)	7.35 (0–10)	12.20 (0–10)	0.809
Need for hemodialysis	23 (31.0%)	57 (28.6%)	0.765
ICU length of stay	13.08 (8.0–18.2)	16.91 (4.4–29.4)	0.035
28-day mortality	44 (59.4%)	105 (52.7%)	0.341
90-day mortality	35 (47.2%)	69 (34.6%)	0.039
Total mortality	38 (51.3%)	136 (68.3%)	0.023

Note: All categorical variables are expressed as numbers and percentages; continuous variables are presented as median interquartile range. Median values and interquartile ranges are presented for non-normally distributed variables. Days without vasopressor support were defined as the number of days within the ICU stay during which the patient was alive and not receiving vasopressors (median, IQR). Abbreviations: ICU: Intensive care unit; CPR: Cardiopulmonary resuscitation; IQR: Interquartile range.

Regarding long-term outcomes, the 90-day mortality rate was significantly higher in the early admission group (47.2%) compared to the delayed admission group (34.6%) (*P* ═ 0.039) (Table 2). No significant difference was noted for 28-day mortality (*P* ═ 0.341). The overall in-hospital mortality rate was 63.7%. Among female patients, the overall in-hospital mortality was 56.9%, with 28-day mortality at 60.2% and 90-day mortality at 46.3% (Table 1). In multivariate Cox regression analyses, delayed admission was not independently associated with either 28-day or 90-day mortality. Instead, hospitalization diagnosis and comorbidity burden, as measured by the Charlson Comorbidity Index, emerged as significant predictors (Table 3).

**Table 3:** Multivariate Cox regression analysis of predictors of 28-day and 90-day mortality

*Model 1*
	**28-day HR**	**95% CI**	**90-day HR**	**95% CI**
Gender (male)	1.143	0.979–1.639	1.188	0.864–1.663
Age	1.001	0.988–1.014	1.004	0.992–1.016
Charlson comorbidity index	1.059	0.990–1.132	1.032	0.971–1.096
Comorbidities	0.748	0.493–1.135	0.958	0.664–1.382
Hospitalization diagnosis (respiratory and circulatory failure)	2.170	1.509–3.122	2.099	1.525–2.889
Late admission (>6 h)	0.948	0.627–1.433	0.920	0.634–1.335
*Model 2*				
	**28-day HR**	**95% CI**	**90-day HR**	**95% CI**
Gender (male)	1.146	0.799–1.643	1.152	0.839–1.583
Charlson comorbidity index	1.064	1.006–1.126	1.059	1.007–1.114
Late admission (>6 h)	1.054	0.697–1.592	0.924	0.638–1.338

Note: Hazard ratios (HR) and 95% confidence intervals (CI) are shown for each model. Abbreviations: HR: Hazard Ratio; CI: Confidence Interval.

In the subgroup analysis of female patients, delayed ICU admission was linked to significantly lower GCS scores and higher Acute Physiology and Chronic Health Evaluation II (APACHE-II) scores at baseline. However, no significant differences were observed in ICU length of stay, vasopressor-free days, or mortality outcomes (Table S2).

Admission diagnoses related to respiratory and circulatory failure were strongly correlated with an increased risk of mortality (HR for 28-day mortality: 2.170, 95% CI: 1.509–3.122) (Table 3). Additionally, higher Charlson Comorbidity Index scores were associated with increased mortality in both multivariate models (Model 1 and Model 2, HR: 1.057–1.064) (Table 3).

Spearman correlation analysis indicated that lower GCS scores and higher APACHE-II and Charlson Comorbidity Index values were significantly associated with increased delays in ICU admission (*P* < 0.01, not shown in tables).

### Secondary results

Delayed admission was significantly associated with a reduction in mechanical ventilation-free days (median 0 vs 1 day; *P* ═ 0.003), while it correlated with an increase in vasopressor-free days (median 0 vs 10 days; *P* ═ 0.809), indicating a complex pattern of resource utilization (Table 2).

Survival analysis based on ICU admission waiting time revealed that patients admitted after more than 4 h had significantly lower survival rates (33.04%) compared to those admitted within 2 h (53.3%); however, this difference did not achieve statistical significance (*P* ═ 0.060) (Table 4).

**Table 4:** Relationship between waiting time for ICU admission and patient mortality

**Length of waiting for ICU admission**	**Survivor *n* (%)**	**Death *n* (%)**	***P* value**
≤ 2 h	8 (53.3%)	7 (46.7%)	
2–4 h	13 (52%)	12 (48%)	
> 4 h	77 (33.04%)	156 (66.96%)	
Total	98 (35.89%)	175 (64.11%)	0.060

Note: Mortality rates are shown for categorized ICU waiting times. Abbreviation: ICU: Intensive care unit.

There was no statistically significant difference in mortality rates between on-duty and off-duty admissions (*P* ═ 0.267) (Table 5), suggesting consistent care delivery irrespective of the time of admission.

**Table 5:** Mortality and survival rates of patients admitted during on-duty and off-duty hours

**Admission time**	**Non-survivors *n* (%)**	**Survivor *n* (%)**	**Total**	***P* value**
Off-duty (17.00–08.00)	81 (29.7%)	53 (19.4%)	134 (49.1%)	
On-duty (08.00–17.00)	93 (34%)	46 (16.9%)	139 (50.9%)	
Total	174 (63.7%)	99 (36.3%)	273 (100%)	0.267

Note: Overall survival compared according to time of ICU admission. Abbreviation: ICU: Intensive care unit.

Baseline demographic analysis indicated no significant age differences between groups (*P* ═ 0.821), although older patients were more likely to experience delays. GCS scores were significantly lower in the delayed admission group (median 3 vs 14; *P* < 0.001), suggesting that patients with more severe neurological compromise faced longer delays in ICU admission. Furthermore, respiratory failure as a reason for ICU admission was more prevalent in the early admission group (*P* ═ 0.002) (Table 2). No significant differences were noted in comorbidity profiles or the need for hemodialysis (Tables 1 and 2).

## Discussion

This study investigated the impact of delayed ICU admission on resource utilization and patient outcomes, focusing primarily on ICU length of stay and long-term mortality. Although delayed ICU admission was not an independent predictor of mortality in multivariate analyses, it was significantly associated with prolonged ICU stays and increased 90-day mortality in univariate analyses. Our findings indicate that delayed ICU admission leads to increased resource utilization and worsens long-term outcomes.

While early ICU admission was linked to significantly higher 90-day mortality in univariate analyses (47.2% vs 34.6%, *P* ═ 0.039), the adjusted hazard ratio in multivariate Cox models was less than 1 and did not achieve statistical significance. This paradox may be explained by confounding variables included in the model. Specifically, patients with early admissions tended to be older and had higher comorbidity burdens and illness severity scores, all of which were adjusted for in the multivariate analysis. These adjustments may have diminished the independent impact of admission timing on mortality. The loss of statistical significance observed in adjusted models suggests that confounding factors related to comorbidity and severity may have influenced the findings, indicating that sicker patients were more likely to be admitted earlier. Additionally, selection bias may have contributed, as patients perceived to have better short-term prognoses might have been deferred and admitted later. Consequently, the lower hazard ratios observed in adjusted models should be interpreted cautiously, as they reflect the complex interplay of multiple clinical variables.

Our findings reveal that patients with greater disease burdens—indicated by elevated APACHE-II and Charlson Comorbidity Index scores, along with reduced levels of consciousness—were more frequently subjected to delays in ICU admission. This pattern raises critical concerns regarding triage dynamics, where sicker patients may paradoxically experience longer waits for ICU access, potentially due to system overload or the prioritization of patients perceived to have better prognoses [5].

The lack of association between admission delay and 28-day mortality suggests that short-term survival is primarily determined by baseline physiological status. However, the observed increase in 90-day mortality among patients with delayed ICU admission aligns with existing literature linking admission delays to adverse long-term outcomes, particularly in those with high illness severity [12, 13]. These results indicate that the consequences of delayed ICU care extend beyond initial stabilization and may adversely affect functional recovery and post-ICU survival.

Importantly, sex-stratified analyses demonstrated that female patients, accounting for 45% of the cohort, presented with slightly higher GCS scores and lower APACHE-II scores at admission compared to males, despite similar comorbidity burdens. In women, delayed ICU admission was associated with fewer ventilator-free days and markers of greater illness severity; however, no significant differences were observed in ICU length of stay or mortality between early and delayed admission subgroups (Tables 1 and 2). These findings suggest that the adverse long-term mortality signal associated with delayed ICU admission in the overall cohort was less pronounced among female patients.

Furthermore, we found that delayed admission significantly prolonged the duration of mechanical ventilation. Although the late-admission group appeared to have more vasopressor-free days, this difference was not statistically significant and likely reflects residual confounding rather than a true protective effect. Consequently, no consistent association was observed between admission delay and vasopressor requirement [14–16]. While our primary analysis utilized a binary classification of ICU admission delay consistent with prior literature, the additional stratification into ≤2 h, 2–4 h, and >4 h in Table 4 was conducted post hoc and intended for exploratory purposes only. We acknowledge that such data-driven cut points may inflate the risk of type I error and obscure potential dose–response relationships. Future studies should consider modeling ICU waiting time as a continuous variable using spline functions to detect potential nonlinear effects. Modeling ICU waiting time in this manner—using restricted cubic splines or generalized additive models—may preserve more statistical information and allow for a more robust assessment of non-linear effects. We recommend that future studies adopt such approaches for a more nuanced analysis of this relationship.

The delayed admission rate in our study (72.8%) was notably higher than that reported in studies from Israel, France, the UK, and Hong Kong [17–20], likely reflecting system-level differences in ICU capacity, referral logistics, and hospital organization. This underscores an urgent need for national strategies to improve coordination and reduce avoidable delays in ICU access. The relatively high rate of delayed ICU admissions (72.8%) in our cohort may appear as an outlier when compared to data from Western countries; however, it reflects the real-world conditions of many tertiary care hospitals in the Middle East and low-to-middle-income countries, where ICU bed shortages, high patient volumes, and limited critical care resources frequently contribute to extended waiting times for ICU admission. Our findings are therefore representative of regional healthcare challenges and provide valuable insight into system-level barriers to timely critical care delivery.

Interestingly, no significant difference in mortality was observed between on-duty and off-duty admissions, contrary to earlier findings [21, 22]. This may reflect the presence of robust staffing models and consistent clinical standards in our institution. Overall, our findings support the need for institutional protocols that prioritize early ICU access for high-risk patients and streamline triage processes to prevent avoidable delays. In resource-limited settings, enhancing ICU workflow efficiency may be as critical as increasing bed numbers.

### Limitations

This study has several notable limitations. First, the retrospective single-center design restricts the generalizability of the findings and may introduce selection bias. Second, variables such as age, Charlson Comorbidity Index (CCI), and the number of comorbidities are closely related and may have introduced multicollinearity. However, VIF diagnostics indicated no significant multicollinearity (all VIF values < 2.5). Additionally, modeling CCI as a continuous linear variable may inadequately capture non-linear associations with mortality at higher comorbidity levels.

Third, we acknowledge that baseline severity and prognosis are critical confounders. In our analysis, we attempted to adjust for severity using the APACHE-II score, CCI, and GCS scores in the multivariate models; however, residual confounding cannot be entirely excluded. Patients with extremely poor prognoses may have influenced the observed associations, particularly concerning long-term mortality and ICU resource utilization, necessitating cautious interpretation of our findings. Furthermore, we did not conduct condition-matched subgroup analyses (e.g., within specific diagnostic or age strata), which may further limit the generalizability of our results.

Fourth, using in-hospital mortality as a survival endpoint without standardized censoring may not fully reflect the time-dependent nature of outcomes. Moreover, more advanced modeling techniques—such as penalized regression or spline analysis—were not employed due to sample size and resource constraints. Finally, detailed data on chronic outpatient medication use, including cardioprotective agents such as SGLT2 inhibitors and GLP-1 receptor agonists, were not consistently available in the medical records. Consequently, we were unable to evaluate the potential influence of these medications on cardiovascular outcomes or ICU admission risk in patients with diabetes mellitus, heart failure, or chronic kidney disease.

## Conclusion

Although early ICU admission was not identified as an independent predictor of mortality in multivariate analysis, it was significantly associated with prolonged ICU stays, increased treatment burdens, and higher unadjusted 90-day mortality rates. These findings suggest that early admission may adversely affect long-term outcomes and resource utilization in critically ill patients. However, due to the retrospective nature of the study, potential confounding factors, and limitations in modeling strategies, these associations should be interpreted with caution. Rather than indicating causality, our findings reflect patterns observed in a high-volume tertiary care ICU, highlighting the need for enhanced triage systems and timely ICU transfers. Prospective, multicenter studies with refined statistical methodologies are necessary to better understand the clinical implications of ICU admission timing.

## Supplemental data

**Table S1:** Schoenfeld residual tests and multicollinearity diagnostics

**Predictor variable**	**Model 1 Tolerance**	**Model 1 VIF**	**Model 2 Tolerance**	**Model 2 VIF**	**Schoenfeld χ^2^**	***P* value**
Age (years)	0.983	1.017	0.985	1.015	0.59	0.44
Gender (0 = Female, 1 = Male)	0.983	1.017	0.985	1.015		
Charlson Comorbidity Index (CCI)	0.565	1.770	0.990	1.010	1.34	0.27
≥3 Comorbidities (yes/no)	0.678	1.475	–	–	0.74	0.39
Late ICU admission (>6h)	0.991	1.009	0.993	1.007	0.82	0.41
Global test (Model 1)	–	–	–	–	7.52	0.47
Global test (Model 2)	–	–	–	–	5.86	0.61

Variable-specific and global Schoenfeld residual test statistics (χ^2^, *P* values) are reported together with variance inflation factor (VIF) and tolerance values for covariates included in the Cox regression models. All *P* values > 0.05 indicate no violation of the proportional hazards’ assumption. All VIF values < 2.0 indicate no problematic multicollinearity. These results indicate no violation of the proportional hazards assumption and no evidence of problematic multicollinearity.

**Table S2:** Female subgroup outcomes by early (≤6 h) versus delayed (>6 h) ICU admission, presented as median (IQR) for continuous variables and *n* (%) for categorical variables

**Variables**	**Early (*n* ═ 38)**	**Late (*n* ═ 85)**	***P* value**
Age, years (median, IQR)	72.5 (63–84)	71 (64–80)	0.590
Glasgow coma scale (median, IQR)	13 (3–15)	3 (3–15)	0.022
Charlson Comorbidity index (median, IQR)	5 (3–7)	6 (4–7)	0.426
Ventilator-free days (median, IQR)	1 (0–3)	0 (0–2)	0.092
Vasopressor-free days (median, IQR)	4 (2–10)	5 (4–9)	0.602
ICU length of stay (median, IQR)	7 (3–14)	9 (4–17)	0.291
APACHE-II score (median, IQR)	19 (13–27)	23 (17–30)	0.035
*Admission diagnosis*			
Respiratory failure, *n* (%)	31 (81.6%)	77 (90.6%)	0.158
Circulatory failure, *n* (%)	18 (47.4%)	40 (47.1%)	0.975
Sepsis, *n* (%)	5 (13.2%)	18 (21.2%)	0.292
Cerebrovascular event, *n* (%)	3 (7.9%)	3 (3.5%)	0.299
Trauma, *n* (%)	1 (2.6%)	1 (1.2%)	0.555
Postoperative, *n* (%)	3 (7.9%)	11 (12.9%)	0.415
CPR, *n* (%)	4 (10.5%)	5 (5.9%)	0.361
Need for hemodialysis, *n* (%)	9 (23.7%)	26 (30.6%)	0.433
28-day mortality, *n* (%)	13 (34.2%)	36 (42.4%)	0.394
90-day mortality, *n* (%)	17 (44.7%)	49 (57.6%)	0.185
Total mortality, *n* (%)	18 (47.4%)	52 (61.2%)	0.153

Note: Continuous variables are reported as median (IQR), tested with Mann–Whitney *U* test. Categorical variables are *n* (%), tested with Pearson chi-square or Fisher’s exact as appropriate. Abbreviations: IQR: Interquartile range; ICU: Intensive care unit; APACHE-II: Acute physiology and chronic health evaluation II; CPR: Cardiopulmonary resuscitation.
